# Short-Chain Fatty Acids Promote Immunotherapy by Modulating Immune Regulatory Property in B Cells

**DOI:** 10.1155/2021/2684361

**Published:** 2021-12-10

**Authors:** Cai-Jie Zhou, Bai-Ling Xie, Hai-Yang Han, Yin Wang, Yong-Hua Wang, Jing-Yi Hong, Yi-Xia Wei, Zhi-Gang Liu, Yan Feng, Gui Yang, Ping-Chang Yang

**Affiliations:** ^1^Shenzhen Hospital, Beijing University of Chinese Medicine, Shenzhen, China; ^2^Guangdong Provincial Key Laboratory of Regional Immunity and Diseases, Shenzhen, China; ^3^Institute of Allergy & Immunology, Shenzhen University School of Medicine, Shenzhen, China; ^4^Department of Otolaryngology, Head & Neck Surgery, First Hospital, Shanxi Medical University, Taiyuan, China; ^5^Department of Otolaryngology, Longgang Central Hospital, Shenzhen, China

## Abstract

The dysfunction of regulatory B cells (Breg) may result in immune inflammation such as allergic rhinitis (AR); the underlying mechanism is not fully understood yet. Short-chain fatty acids, such as propionic acid (PA), have immune regulatory functions. This study is aimed at testing a hypothesis that modulates PA production alleviating airway allergy through maintaining Breg functions. B cells were isolated from the blood obtained from AR patients and healthy control (HC) subjects. The stabilization of IL-10 mRNA in B cells was tested with RT-qPCR. An AR mouse model was developed to test the role of PA in stabilizing the IL-10 expression in B cells. We found that the serum PA levels were negatively correlated with the serum Th2 cytokine levels in AR patients. Serum PA levels were positively associated with peripheral CD5^+^ B cell frequency in AR patients; the CD5^+^ B cells were also IL-10^+^. The spontaneous IL-10 mRNA decay was observed in B cells, which was prevented by the presence of PA through activating GPR43. PA counteracted the effects of Tristetraprolin (TTP) on inducing IL-10 mRNA decay in B cells through the AKT/T-bet/granzyme B pathway. Administration of Yupinfeng San, a Chinese traditional medical formula, or indole-3-PA, induced PA production by intestinal bacteria to stabilize the IL-10 expression in B cells, which promoted the allergen specific immunotherapy, and efficiently alleviated experimental AR. In summary, the data show that CD5^+^ B cells produce IL-10. The serum lower PA levels are associated with the lower frequency of CD5^+^ B cells in AR patients. Administration with Yupinfeng San or indole-3-PA can improve Breg functions and alleviate experimental AR.

## 1. Introduction

The immune regulation indicates a balance between activation and suppression of effector immune cells to achieve an efficient immune response without causing damage in the host that is carried out by a group of immune cells. This group of cells is designated immune regulatory cells, such as, but not limited to, regulatory T cells (Treg) and regulatory B cells (Breg) [1, 2]. Examples of immune regulatory cytokines are IL-10 and transforming growth factor- (TGF-) *β*. It is recognized that the immune regulatory cell number reduction or/and dysfunction is associated with the pathogenesis of immune diseases [1, 2]. Thus, maintaining immune regulatory cells at the optimal functional status may help to generate new therapies for immune diseases.

Upon proper activation, Bregs release immune regulatory cytokines, mainly IL-10, to suppress other immune cell activities that restrict immune responses within a proper range to avoid unnecessary injuries to tissues or/and cells [2]. Several cell markers have been described for Bregs, such as CD5, CD34, CD73, CD71, or CD25, indicating that Breg markers and functions may be modified by relevant environmental factors [3], one of which is the short-chain fatty acids (SCFAs) [4]. SCFAs are metabolites of intestinal microbiota on fermenting dietary fibers that can regulate differentiation or functions of immune cells. Propionic acid (PA) is one of the SCFAs, which has been shown to induce or promote the immune regulatory capacity of CD25^+^ Foxp3^+^ Treg and Breg [4, 5]. Yet, the underlying mechanism is not fully understood yet.

Allergic diseases, such as allergic rhinitis (AR), asthma, and dermatitis, are common immune disorders. The major immune pathological feature of allergic diseases is the Th2 polarization. Th2 polarization indicates a condition that the local tissues are overpopulated by Th2 cells and the local tissues are oversaturated with Th2 cytokines, which can be reflected in the blood stream, and is detectable by enzyme-linked immunosorbent assay (ELISA) [6]. On the other hand, the Th2 polarization indicates a dysfunctional status of the immune regulatory system in the body. The causative factors and underlying mechanism of immune regulatory dysfunction remain to be further investigated. Based on the above information, we hypothesize that modulating PA production can alleviate airway allergy through maintaining Breg functions. In this study, we observed that the IL-10 mRNA spontaneously decayed in Bregs, which played a critical role in the Breg dysfunction. The presence of PA or administration of Yupinfeng San (YPS), a Chinese traditional medicine formula that showed positive effects on suppressing AR in clinic and animal model studies [7–9], promoted the production of PA by intestinal bacteria to prevent the IL-10 mRNA decay and alleviated experimental AR.

## 2. Materials and Methods

### 2.1. Human Subjects

Patients with AR were recruited into the present study at the Longgang Chinese Traditional Hospital (Shenzhen, China), Longgang Central Hospital (Shenzhen, China), and Longgang ENT Hospital (Shenzhen, China) from June 2020 to June 2021. The diagnosis of AR was carried out by our doctors following the routine procedures of the hospitals that also can be found elsewhere [10]. The demographic data are presented in Table 1. Patients with any of the following conditions were excluded: cancer, autoimmune diseases, severe organ diseases, and undergoing treatment of immune suppressors or corticosteroids for any reasons. Healthy control (HC) subjects were also recruited in the hospitals. Blood samples were collected from 36 AR patients and 36 healthy control (HC) subjects. The serum was separated from the blood samples and analyzed by high-performance liquid chromatography (HPLC) and ELISA. The experimental procedures were approved by the Human Ethical Committee at the Longgang Chinese Traditional Hospital (approve#2019HE015), Longgang ENT Hospital, and Longgang Central Hospital (approve#2019HE0030). A written informed consent was obtained from each human subject.

### 2.2. Development of an AR Mouse Model and Specific Immunotherapy (SIT)

BALB/c mice (6-8 weeks old) were purchased from the Guangzhou Experimental Animal Center. Mice were maintained in a specific pathogen-free facility with accessing food and water freely. The animal experimental procedures were approved by the Animal Ethical Committee at Shenzhen University (approve#2019035). Mice were sensitized to ovalbumin (OVA) following established procedures [11], which is also depicted in Fig. S1 in the supplemental materials. AR clinical symptoms (nasal itch and sneezing) was observed and recorded during 30 min after the nasal challenge. The nasal lavage fluids (NLFs) were then collected by introducing 30 *μ*l saline into each nostril, and the NLF was immediately collected with the same syringe. The rinse was repeated 3 times. All NLFs were pooled and analyzed by ELISA. A group of mice was gavage-fed with indole propanoic acid (IPA; 20 mg/kg/day) or vehicle once daily during the sensitization. For SIT, right before each time of nasal instillation with OVA, mice were sublingually administered with 1 mg of OVA in 20 *μ*l of PBS during sensitization. To prevent immediate swallowing of the allergen, mice were held on the back for 1 min [12].

### 2.3. Isolation of CD5^+^ B Cells in Mouse Airway Tissues

After the sacrifice, the nasal tissues and lungs were excised, cut into small pieces, incubated with collagenase IV (0.5 mg/ml) for 30 min at 37°C with mild agitation to dissociate airway mononuclear cells (AMC). AMCs were filtered through a cell strainer (100 *μ*m first, then 40 *μ*m) and centrifuged at 1000 g for 5 min. The pellets were resuspended and washed with PBS, stained with fluorescence-labeled antibodies of CD19 and CD5, and analyzed by flow cytometry (FACS).

### 2.4. Depletion of Intestinal Bacteria

Mice were gavage-fed with vancomycin (50 mg/kg), neomycin (100 mg/kg), metronidazole (100 mg/kg), and amphotericin-B (1 mg/kg) every 12 hours with ampicillin 1 g/l ad libitum in drinking water for 7 consecutive days. Control mice received water ad libitum only. To determine the effects of bacterial depletion, mouse fecal culture was performed following published procedures [13]. The results showed that less than 1 cfu/mg feces in mice treated with gavage-feeding with antibiotics.

### 2.5. Statistics

The differences between two groups were determined by Student *t* test or Mann-Whitney *U* test. ANOVA followed by Dunnett's test was performed for multiple comparisons. Spearman correlation coefficient test was performed for determining correlation between two datasets. *p* < 0.05 was set as a significant criterion.

## 3. Results

### 3.1. Serum PA Levels Are Negatively Correlated with Serum Th2 Cytokine Levels in AR Patients

**Figure 1:**
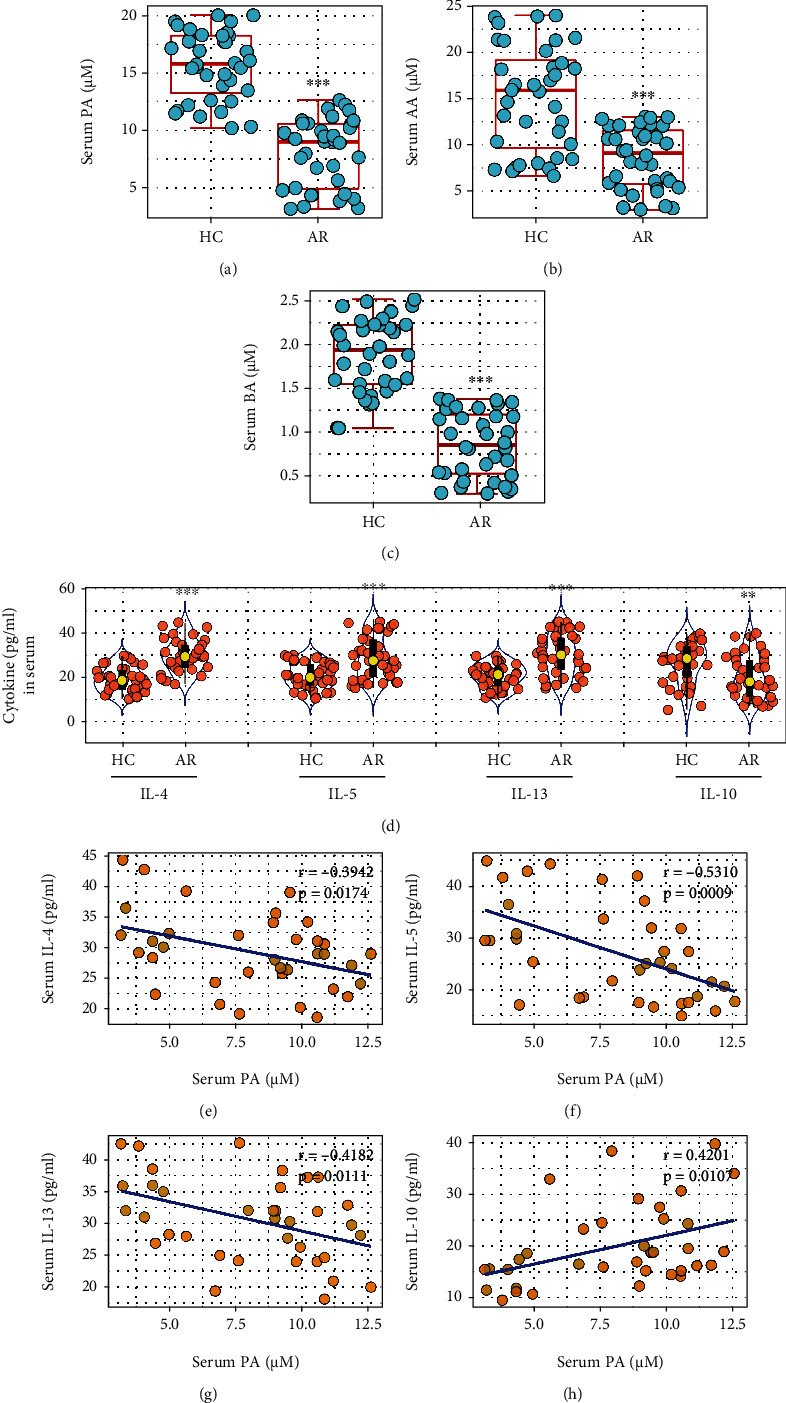
Serum PA negatively correlates with human serum Th2 cytokines. Blood samples were collected from 36 AR patients and 36 healthy control (HC) subjects. The serum was separated from blood samples and analyzed by HPLC and ELISA. (a–c) Boxplots show the serum levels of propionic acid (PA), acetic acid (AA), and butyric acid (BA). (d) Violin plots show serum Th2 cytokine levels. (e–g) Negative correlation between serum PA, serum Th2 cytokines, and IL-10 of the AR group (determined by Spearman correlation coefficient test). ^∗∗^*p* < 0.01 and ^∗∗∗^*p* < 0.001 (Mann-Whitney *U* test), compared with the HC group. Each dot presents data obtained from one sample.

We found that the serum levels of acetic acids (AA), butyric acids (BA), and propionic acids (PA) were lower in the AR group than in the HC group (Figures 1(a)–1(c)). Serum Th2 cytokine (including IL-4, IL-5, and IL-13) levels were higher, and IL-10 levels were lower in the AR group as compared with the HC group (Figure 1(d)), indicating a Th2 polarization status in AR patients. A negative correlation was detected in the data between serum PA levels and serum Th2 cytokine levels (Figures 1(e)–1(g)), while the serum IL-10 levels were positively correlated with serum PA levels (Figure 1(h)). The serum Th2 cytokine levels and IL-10 levels did not show correlation with either serum AA or BA (Fig. S2 in supplemental materials). The data suggest that the lower serum PA levels may be associated with the pathogenesis of Th2 polarization in AR.

### 3.2. Serum PA Levels Are Positively Associated with Peripheral CD5^+^ B Cell Frequency

**Figure 2:**
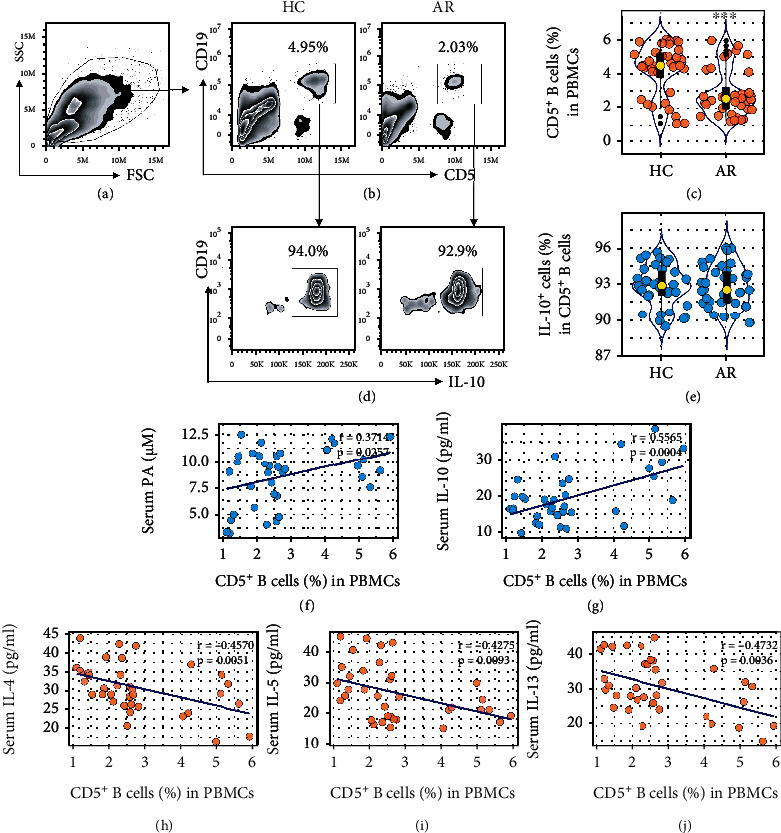
A negative correlation between human peripheral CD5^+^ B cells and serum Th2 cytokines. (a–c) Blood samples were collected from 36 AR patients and 36 HC subjects. PBMCs were analyzed by FACS. (a) The FSC/SSC plots. (b) Gated plots show CD5^+^ B cells. (c) Violin plots show CD5^+^ B cell frequency in PBMCs. (d) Gated plots show IL-10^+^ cells in CD5^+^ B cells. (e) Violin plots show IL-10^+^ cell frequency in CD5^+^ B cells. (f, g) A positive correlation between CD5^+^ B cell frequency and serum PA levels or serum IL-10 levels (determined by Spearman correlation coefficient test). (h–j) A negative correlation between B10 cell frequency and serum Th2 cytokines. ^∗∗∗^*p* < 0.001 (Mann-Whitney *U* test), compared with the HC group.

As CD5^+^ B cells produce IL-10, and are associated with oral tolerance [14], Th2 polarization usually implicates the immune tolerance disruption [2]; we next assessed the peripheral CD5^+^ B cell frequency [14] in AR patients. The results showed that CD5^+^ B cells were detectable in PBMCs that were fewer in the AR group than in the HC group (Figures 2(a)–2(c)); more than 90% CD5^+^ B cells were also IL-10^+^ (Figures 2(d) and 2(e)). The CD5^+^ B cells showed immune suppressive effects on T cell proliferation (Fig. S3). A positive correlation was detected between the CD5^+^ B cell frequency and the serum PA levels or IL-10 levels (Figures 2(f) and 2(g)), implicating that PA may involve CD5^+^ B cell activities, and the latter is associated with the serum IL-10 levels. In addition, a negative correlation between the CD5^+^ B cell frequency and the serum Th2 cytokines was detected (Figures 2(h)–2(j)). The results suggest that the serum PA levels may be associated with the immune tolerance disruption in AR patients since CD5^+^ B cells are one of the major immune-tolerant cell fractions [14–16].

### 3.3. PA Prevents IL-10 mRNA Decay in B Cells

**Figure 3:**
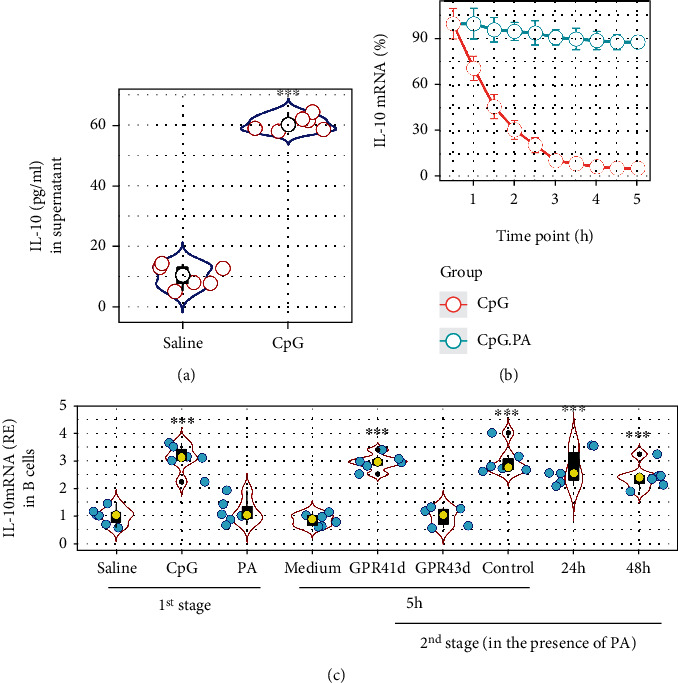
PA stabilizes IL-10 mRNA in human B cells. B cells were isolated from blood samples collected from HC subjects. (a) Violin plots show IL-10 protein levels in culture supernatant after exposing to CpG (1 *μ*g/ml) for 16 h (the 1^st^ stage). (b) The curve shows IL-10 mRNA levels in B cells after a 5 h culture (the 2^nd^ stage) in fresh medium in the presence or absence of PA following the exposure to CpG in the culture for 16 h. (c) Violin plots show IL-10 mRNA levels in B cells after the treatment denoted on the *x* axis at different time points. PA: 10 *μ*M; GPR41d (GPR43d): GPR41-deficient (or GPR43-deficient) B cells (prepared by RNAi); Control: B cells were treated with scrambled RNAi. ^∗∗∗^*p* < 0.001 (*t* test for (a); ANOVA+Dunnett's test for (c)), compared with the saline group. Each dot in the violin plots presents data obtained from one experiment.

It is the consensus that the IL-10 expression is the mainstay of regulatory B cell function. We then assessed the association between PA and the capacity of IL-10 production by B cells. Naïve B cells were isolated from blood samples collected from HC subjects. As B cells do not spontaneously produce IL-10, CpG (an IL-10 inducer) was added to the culture. We found that exposure to CpG in the culture for 16 h induced the IL-10 expression (Figure 3(a)). Following our established procedures [17], the B cells were washed and cultured in fresh medium without the presence of CpG. We found that the IL-10 expression in B cells declined 30 min after, which progressively reduced to the baseline levels 5 h later. The results indicate that IL-10 mRNA decays spontaneously soon after splicing. Prompted by the results of Figures 1 and 2, we speculated that PA might counteract the IL-10 mRNA decay in B cells. To test this, we repeated the experiments with the addition of PA to the culture. Indeed, the presence of PA efficiently counteracted the IL-10 mRNA decay (Figure 3(b)).

Both GPR41 and GPR43 can be recognized by PA. To elucidate which one involves the PA-mediated IL-10 mRNA stabilization in B cells, we prepared GPR41-deficient (GPR41d) and GPR43d B cells by RNA interference (RNAi) (Fig. S4). Then, wild-type (WT) B cells, GPR41d B cells, and GPR43d B cells were exposed to CpG in the culture for 16 h and washed with medium, and the culture was continued with fresh medium (containing PA) for another 5 h. The B cells were harvested at the end of the culture and analyzed by RT-qPCR. The results showed that the IL-10 mRNA expression was maintained by the presence of PA in the WT group and the GPR41d group but went down to the baseline levels in the GPR43d group (Figure 3(c)). The results demonstrate that GPR43 mediates the effects of PA on regulating the IL-10 expression in B cells. In addition, we extended the culture time to 24 h and 48 h in the second stage, respectively. We found that the IL-10 expression was still maintained in B cells by the presence of PA (Figure 3(c)). The results indicate that the presence of PA counteracts the spontaneous IL-10 mRNA decay in B cells.

### 3.4. PA Counteracts the Effects of Tristetraprolin (TTP) on Inducing IL-10 mRNA Decay in B Cells

**Figure 4:**
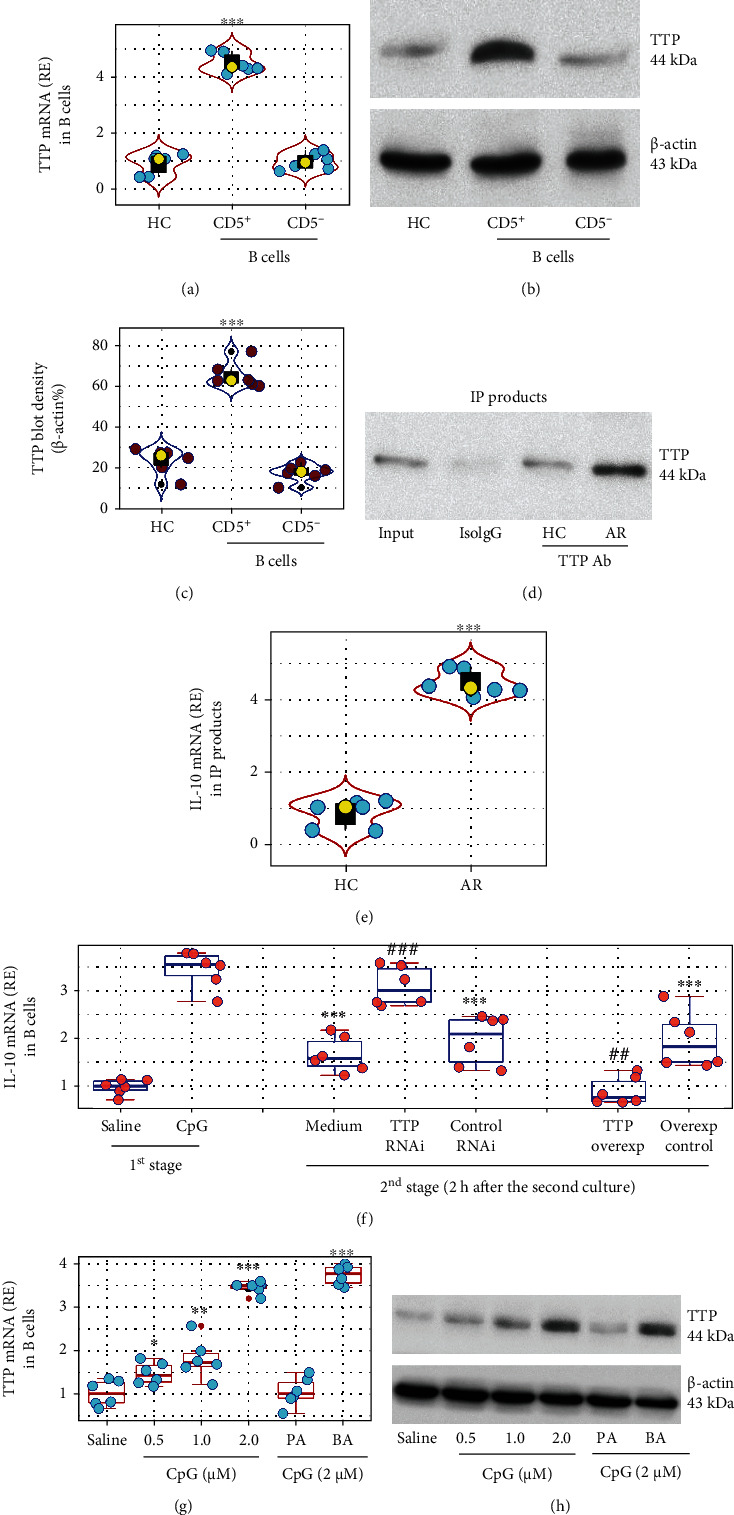
TTP forms complexes with IL-10 mRNA in human B cells. (a–c) CD5^+^ and CD5^−^ B cells were isolated from blood samples collected from HC subjects and AR patients. Violin plots show TTP mRNA (a), and immunoblots show TTP protein (TTP) levels in B cells. Boxplots show the integrated density of TTP immunoblots (c). (d, e) IP products with an anti-TTP Ab as a bait. Violin plots show IL-10 mRNA in the IP products, indicating that TTP forms a complex with IL-10 mRNA. (f–h) B cells were prepared from HC subject blood samples and treated with the methods denoted below the panels. (f) Boxplots show IL-10 mRNA levels in B cells. 1^st^ stage: B cells were cultured overnight in the presence of CpG; 2^nd^ stage: B cells were collected from the 1^st^ stage culture, washed, and cultured in fresh medium for 1 h; TTP RNAi: B cells were treated with TTP RNAi; Control RNAi: B cells were treated with control RNAi; TTP overexp: B cells were transfected with TTP expressing plasmids; Overexp control: B cells were treated with empty plasmids. (g, h) B cells were cultured overnight with the treatment denoted below the plots. PA: 10 *μ*M in the culture; BA: 2 *μ*M in the culture. Boxplots show TTP mRNA levels in B cells. Immunoblots show TTP protein levels in B cells. ^∗^*p* < 0.05, ^∗∗^*p* < 0.01, ^∗∗∗^*p* < 0.001, compared with the HC group (a, c, e), the CpG group (f), or the saline group (g). ^##^*p* < 0.01 and ^###^*p* < 0.001, compared with the medium group. Statistical methods: Student *t* test (e) and ANOVA+Dunnett's test (a, c, f, g).

Our previous studies showed that TTP bound IL-10 mRNA to induce IL-10 decay in B cells of an experimental food allergy study, but the underlying mechanism remains to be further investigated [18]. In line with this, we also found that CD5^+^ B cells, but not CD5^−^ B cells, collected from AR patients showed higher TTP expression (Figures 4(a)–4(c)). By immunoprecipitation assay with an anti-TTP antibody as bait, we precipitated TTP protein (Figure 4(d)). IL-10 mRNA was detected in the IP products of TTP, which was significantly higher in the AR samples (Figure 4(e)). The IL-10 mRNA decay in B cells was blocked by knocking down the TTP expression (Figure 4(f), Fig. S5A). Overexpression of TTP in B cells (Fig. S5B) sped up the IL-10 mRNA decay in B cells (Figure 4(f)). This phenomenon was reproduced by exposing B cells to CpG in the culture that was abrogated by the presence of PA, but not by BA (Figures 4(g) and 4(h)). The results indicate that TTP forms a complex with IL-10 mRNA and induces the IL-10 mRNA decay in B cells, which can be counteracted by the presence of PA.

### 3.5. PA Inhibits TTP in B Cells through the AKT-T-bet-GZMB Pathway

**Figure 5:**
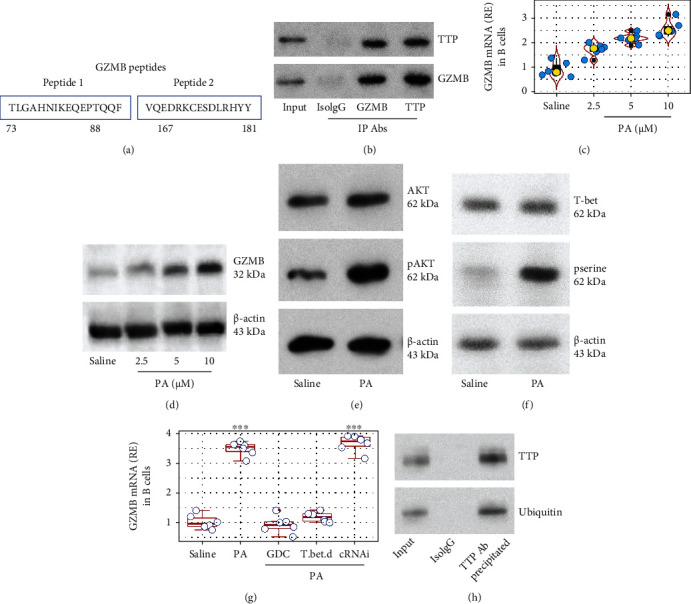
PA induces TTP degradation through the AKT/T-bet pathway. (a) Naive human B cells were treated with CpG and PA in the culture overnight. Protein extracts of B cells were precipitated with an anti-TTP Ab. The IP products were analyzed by mass spectrometry (MS). The amino acid sequences are two representative peptides of GZMB amino acid sequence identified by MS. (b) Immunoblots show a complex of TTP and GZMB in IP products. (c–f) Naïve B cells were exposed to PA in the culture overnight. (c, d) Violin plots show GZMB mRNA, and immunoblots show GZMB protein levels in the B cells. (e, f) Immunoblots show phosphor AKT (e) and T-bet (f) in the B cells. (g) Naïve B cells were treated with the procedures as denoted on the *x* axis. Boxplots show GZMB mRNA levels. GDC: GDC-0068, an AKT inhibitor (5 nM); T.bet.d: T-bet-deficient B cells (prepared by RNAi); cRNAi: B cells were treated with control RNAi; PA: 10 *μ*M. (f) Immunoblots show a TTP/GZMB complex in B cells after the treatment with PA. (g) Immunoblots show the colocalization of TTP and ubiquitin in B cells after treatment with PA. ^∗∗∗^*p* < 0.001 (ANOVA+Dunnett's test), compared with the saline group. Each dot in boxplots presents data obtained from one sample.

We then took further insight into the mechanism by which PA counteracts the effects of TTP on IL-10 mRNA decay. Naïve B cells were treated with CpG and PA in the culture overnight. Protein extracts of the B cells were prepared and analyzed by immunoprecipitation (IP) assay with an antibody of TTP as bait. The IP products were analyzed by mass spectrometry (MS). The MS results showed that granzyme B (GZMB) formed a complex with TTP in B cells after exposure to CpG and PA (Figure 5(a), Fig. S6). The IP products were then analyzed by Western blotting. The results verified the complex of TTP/GZMB in the IP products (Figure 5(b)), indicating that PA induces the GZMB expression in B cells, which was demonstrated by RT-qPCR and Western blotting; PA induced GZMB expression in B cells in a dose-dependent manner (Figures 5(c) and 5(d)). Previous reports indicate that the AKT and T-bet signal pathway is involved in the GZMB expression [19]. We also found this phenomenon in PA-primed B cells, in which the phosphorylation of AKT and T-bet expression was upregulated (Figures 5(e) and 5(f)). The PA-induced GZMB expression in B cells could be abrogated by inhibition of either AKT or T-bet (Figure 5(g)). As GZMB is a protease, we inferred that it might degrade TTP. Indeed, in the IP products of TTP and GZMB, we colocalized TTP and ubiquitin (Figure 5(h)), a sign of TTP degradation [20]. The results indicate that PA inhibits TTP in B cells through the AKT/T-bet/GZMB pathway.

### 3.6. Modulating PA Production Stabilizes IL-10 Expression in B Cells and Suppresses Experimental AR

**Figure 6:**
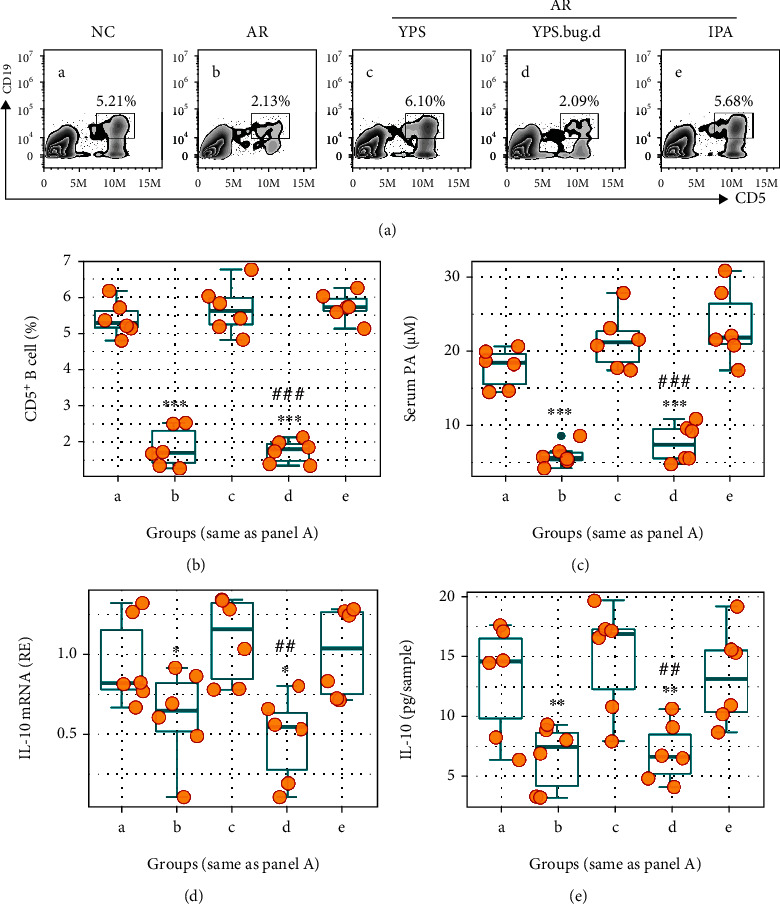
YPS induces PA production by intestinal bacteria to increase CD5^+^ B cells in the mouse airway tissues. AR mice and NC mice were treated with the procedures listed above the FACS plots (a). YPS: mice were gavage-fed with YPS daily during sensitization; Bug.d: mice were gavage-fed with antibiotic cocktail to deplete intestinal bacteria; IPA: mice were fed with indole-PA-3 daily during sensitization. (a) Gated FACS plots show CD5^+^ B cell counts in airway mononuclear cells (AMCs). (b) CD5^+^ B cell frequency in AMCs. (c) Mouse serum PA levels. (d, e) Total RNA and proteins were extracted from CD5^+^ B cells (purified from AMCs and with equal cell number per sample (5 × 10^3^ cells/sample)). Boxplots show IL-10 mRNA levels in airway CD5^+^ B cells. Each group consists of 6 mice. Each dot in boxplots presents data obtained from one sample. ^∗∗∗^*p* < 0.001 (ANOVA+Dunnett's test), compared with the NC group. ^##^*p* < 0.01 and ^###^*p* < 0.001 (*t* test), compared with group c.

YPS is a traditional Chinese medicine formula. Previous studies indicate that YPS can alleviate AR and asthma by restoring the Breg function [7–9]. We hypothesize that such an effect of YPS may be through inducing PA production by intestinal bacteria, stabilizing IL-10 mRNA in B cells. To test this, a murine AR model was developed with established procedures [11]. The CD5^+^ B cell frequency and serum PA levels were lower in AR mice, which were significantly increased in mice treated with YPS, but did not occur in mice with depleted bacteria (Figures 6(a)–6(c)). The IL-10 expression in CD5^+^ B cells of the airway tissues was diminished in the AR group, which was upregulated by the administration of YPS (Figures 6(d) and 6(e)).

**Figure 7:**
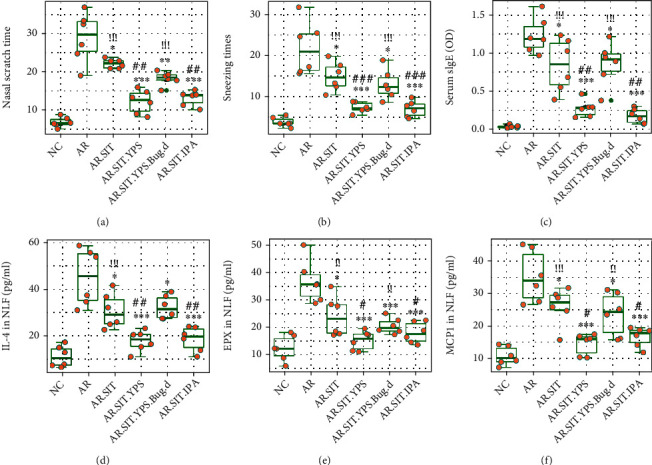
Modulating PA production promotes SIT effects in mice. AR mice were treated with the procedures listed on the *x* axis. NC: naïve control mice; AR: AR mice; SIT: AR mice treated with SIT; YPS: mice treated with YPS; Bug.d: mice treated with antibiotics to deplete intestinal bacteria; IPA: mice treated with IPA. (a, b) AR symptoms of mice, including nasal scratch times (nasal itch) (a) and sneezing (b). (c) Serum sIgE levels. (d–f) Levels of IL-4 (d), EPX (e), and MCP1 (mast cell protease-1) (f) in nasal lavage fluids (NLF). Each group consists of 6 mice. Each dot in boxplots presents data obtained from one mouse. ^∗^*p* < 0.05, ^∗∗^*p* < 0.01, and ^∗∗∗^*p* < 0.001, compared with the AR group. ^#^*p* < 0.05, ^##^*p* < 0.01, and ^###^*p* < 0.001, compared with the AR.SIT group. ^!!^*p* < 0.01 and ^!!!^*p* < 0.001, compared with the NC group. Statistical method: ANOVA+Dunnett's test.

The AR response, including AR symptoms (nasal itch and sneezing), serum sIgE, allergic mediators, and cytokines in nasal lavage fluids (NLF), was partially alleviated in AR mice treated with SIT or YPS, while significantly alleviated by combination of SIT and YPS or SIT and IPA. The alleviating effects were abolished by depleting intestinal bacteria with antibiotics (Figure 7). Depletion of intestinal bacteria alone did not show any effects on AR response (data not shown). Taking together the results in Figures 6 and 7, modulating PA production stabilizes the IL-10 expression in Bregs, which can promote SIT efficacy in the treatment of allergic diseases such as AR.

## 4. Discussion

The present paper reports a previously undescribed phenomenon that PA stabilizes the IL-10 expression in B cells via activating the AKT-T-bet pathway. We found that the serum PA levels were lower in AR patients, which were negatively correlated with the Th2 cytokine levels in the serum. We also found that the serum PA levels were positively correlated with peripheral CD5^+^ B cells, a fraction of B cells expressing IL-10. We further found that PA could counteract the IL-10 mRNA decay in B cells through the AKT-T-bet pathway. Administration of YPS induced intestinal bacteria to produce PA that stabilized peripheral CD5^+^ B cells and alleviated experimental AR.

In line with published data [14], we also found that CD5^+^ B cells expressed IL-10. This fraction of B cells was much fewer in AR patients. Since the expression of IL-10 is the mainstay in Bregs, this fraction of IL-10-expressing CD5^+^ B cells therefore belongs to Bregs. The major beneficial function of Breg is to suppress the aberrant immune activities of other immune cells through acting in concert with T cells [21]. Abnormal B cell activities are associated with the pathogenesis of many autoimmune diseases [22]. Our data are consistent with these studies by showing that IL-10^+^ CD5^+^ B cell frequency is lower in AR patients, suggesting that the abnormality of this fraction may contribute to the pathogenesis of AR.

In parallel to the CD5^+^ B cell frequency, the serum IL-10 levels are lower in the AR group as compared with the HC group. The positive correlation between the CD5^+^ B cell frequency and serum IL-10 levels suggests that CD5^+^ B cells are the important source of serum IL-10 in the present experimental setting. Thus, to understand the mechanism by which IL-10 decreased in B cells is of importance. We [17, 18] and others [23] previously found that the IL-10 mRNA relatively easily decayed, which degraded spontaneously soon after splicing before completing the IL-10 protein synthesis. Thus, it is necessary to investigate the mechanism of IL-10 mRNA decay. In line with previous reports [17, 18], the present data also show that IL-10 mRNA decayed in B cells soon after the removal of IL-10 inducers and confirm that TTP is the factor to induce IL-10 mRNA decay in B cells.

TTP is an RNA-binding protein. The present data show that TTP binds IL-10 mRNA to form a complex that further induces IL-10 mRNA decay in B cells. By reviewing its amino acid sequence, we found that TTP was a proline-rich molecule. This feature [24] indicates that TTP is prone to bind other molecules. Apart from binding IL-10 mRNA, TTP also binds other molecules' mRNA, such as IL-8 and many others [25] to modulate the posttranscription activities of protein synthesis. Our data show that exposure of B cells to CpG induced both IL-10 mRNA expression as well as TTP expression. This may be understood as a self-regulation activity to maintain the homeostasis in the cell.

The data show that the serum SCFA, including PA, AA, and BA, levels were lower in AR patients as compared to HC subjects. By correlation assay, serum PA, but not AA or BA, levels were negatively correlated with serum Th2 cytokine levels. It has been recognized that PA has immune regulatory functions. The PA intake increases Treg-related gene expression, suppresses proinflammatory Th1 and Th17 cytokines, and alleviates immune diseases, such as multiple sclerosis [26]. Our data show that the presence of PA counteracts the IL-10 mRNA decay in B cells. Because the IL-10 expression is the mainstay in Bregs, the data indicate that PA contributes to maintaining the homeostasis of Bregs.

The present study revealed the mechanism by which PA counteracts the IL-10 mRNA decay in B cells. We found that PA increased the expression of GZMB in B cells; the latter induced TTP degradation. GZMB is a protease that mediates degradation of T cell receptor zeta chain [27], directly induces target cell death [28], and degrades apoptosis inhibitors to promote cell apoptosis [29]. Our data show a signal transduction pathway, AKT/T-bet, by which PA induces GZMB expression in B cells. AKT mediates multiple cell signals that play critical roles in many cellular activities [30]. In line with previous studies that show IL-15 activating the AKT/T-bet signal pathway to elicit GZMB gene transcription [19], we also found that PA activated GPR43 on B cells to elicit the GZMB transcription via the AKT/T-bet pathway.

The data show that lower serum PA levels are negatively correlated with the Th2 polarization and the impaired status of Bregs in AR patients. This suggests that the PA supplement may alleviate AR. Through an animal model study, we found that administration of PA indeed efficiently alleviated AR. On the other hand, a group of AR mice was treated with YPS, a Chinese traditional medicine formula, that demonstrated the effects on inhibition of AR in both the clinic and animal studies [7–9]. The results demonstrate that administration of YPS also can alleviate experimental AR and promote SIT efficacy through increasing PA production by intestinal bacteria and increasing Bregs.

In summary, the present data indicate that PA insufficiency is associated with the pathogenesis of AR. Administration of PA or employing YPS to increase PA can promote SIT efficacy and alleviate experimental AR. The results suggest that PA supplement may have the translation potential to be used in the treatment of AR or other allergic diseases.

## Figures and Tables

**Table 1:** Demographic data of human subjects.

Items	AR patients	HC subjects
Male/female	16/20	17/19
Age (years, median (IQR))	26.5 (16, 48)	25.4 (18, 45)
Specific IgE (median (IQR))	56.8 (38, 115) (kU/L)	5.6 (3.5, 21.6)
*SPT results*		
Bermuda grass	2 (5.6%)	0
Pine	4 (11.1%)	0
Poplar	5 (13.9%)	0
Rye	1 (2.8%)	0
Timothy grass	2 (5.6%)	0
Mugwort	2 (5.6%)	0
Mite mix	36 (100%)	0
Mold mix	8 (22.2%)	0
Animal dander	3 (8.3%)	0

## Data Availability

All the data are included in this paper and the online supplemental materials.

## Supplementary Materials

Supplementary Materials Supplemental materials include reagent information, a portion of experimental information and some results. *Reagents and experimental procedures*: skin prick tests, preparation of peripheral blood mononuclear cells and serum, high-performance liquid chromatography, cell culture, enzyme-linked immunosorbent assay, flow cytometry, isolation of immune cells, Western blotting, real-time quantitative RT-PCR, immunoprecipitation, mass spectrometry, and SIT. *Supplemental figures*: Figure S1: a schematic of mouse airway allergy model development. Figure S2: correlation coefficient between serum Th2 cytokines and serum AA or BA. Figure S3: assessment of immune suppressive effects of CD5^+^ B cells on T cell proliferation. Figure S4: knockdown of GPR41 or GPR43 in B cells. Figure S5: modulating TTP expression in B cells. Figure S6: GZMB amino acid sequence.

## References

[1] Josefowicz S. Z., Lu L. F., Rudensky A. Y. (2012). Regulatory T cells: mechanisms of differentiation and function. *Annual Review of Immunology*.

[2] Rosser E. C., Mauri C. (2015). Regulatory B cells: origin, phenotype, and function. *Immunity*.

[3] Wang R. X., Yu C. R., Dambuza I. M. (2014). Interleukin-35 induces regulatory B cells that suppress autoimmune disease. *Nature Medicine*.

[4] Sanchez H. N., Moroney J. B., Gan H. (2020). B cell-intrinsic epigenetic modulation of antibody responses by dietary fiber-derived short-chain fatty acids. *Nature Communications*.

[5] Smith P. M., Howitt M. R., Panikov N. (2013). The microbial metabolites, short-chain fatty acids, regulate colonic Treg cell homeostasis. *Science*.

[6] Tjota M. Y., Sperling A. I. (2014). Distinct dendritic cell subsets actively induce Th2 polarization. *Current Opinion in Immunology*.

[7] Chan R. Y., Chien W. T. (2014). The effects of two Chinese herbal medicinal formulae vs. placebo controls for treatment of allergic rhinitis: a randomised controlled trial. *Trials*.

[8] Liu X., Shen J., Fan D. (2017). Yupingfeng San inhibits NLRP3 inflammasome to attenuate the inflammatory response in asthma mice. *Frontiers in Pharmacology*.

[9] Zhou C. J., Ma F., Liao W. J. (2019). Restoration of immune suppressor function of regulatory B cells collected from patients with allergic rhinitis with Chinese medical formula Yupingfeng San. *American Journal of Translational Research*.

[10] Wise S. K., Lin S. Y., Toskala E. (2018). International consensus statement on allergy and rhinology: allergic rhinitis. *International Forum of Allergy & Rhinology*.

[11] Zeng X. H., Yang G., Liu J. Q. (2019). Nasal instillation of probiotic extracts inhibits experimental allergic rhinitis. *Immunotherapy*.

[12] Suzuki S., Sakurai D., Sakurai T. (2019). Sublingual administration of liposomes enclosing alpha-galactosylceramide as an effective adjuvant of allergen immunotherapy in a murine model of allergic rhinitis. *Allergology International*.

[13] Reikvam D. H., Erofeev A., Sandvik A. (2011). Depletion of murine intestinal microbiota: effects on gut mucosa and epithelial gene expression. *PLoS One*.

[14] Noh J., Noh G., Lee S. J. (2012). Tolerogenic effects of interferon-gamma with induction of allergen-specific interleukin-10-producing regulatory B cell (Br1) changes in non-IgE-mediated food allergy. *Cellular Immunology*.

[15] van de Veen W., Stanic B., Wirz O. F., Jansen K., Globinska A., Akdis M. (2016). Role of regulatory B cells in immune tolerance to allergens and beyond. *The Journal of Allergy and Clinical Immunology*.

[16] Satitsuksanoa P., Jansen K., Głobińska A., van de Veen W., Akdis M. (2018). Regulatory immune mechanisms in tolerance to food allergy. *Frontiers in Immunology*.

[17] Zhao M., Zeng H. T., Yang G. (2019). Toll-like receptor signal is required in maintenance of immune suppressive capacity of regulatory T cells. *Immunology Letters*.

[18] Zeng H. T., Zhao M., Yang S. B. (2019). Vasoactive intestinal peptide alleviates food allergy via restoring regulatory B cell functions. *Immunobiology*.

[19] Wang Y., Zhang Y., Yi P. (2019). The IL-15-AKT-XBP1s signaling pathway contributes to effector functions and survival in human NK cells. *Nature Immunology*.

[20] Khalil R. (2018). Ubiquitin-proteasome pathway and muscle atrophy. *Advances in Experimental Medicine and Biology*.

[21] Yanaba K., Bouaziz J. D., Haas K. M., Poe J. C., Fujimoto M., Tedder T. F. (2008). A regulatory B cell subset with a unique CD1d^hi^CD5^+^ phenotype controls T cell-dependent inflammatory responses. *Immunity*.

[22] Wu H., Deng Y., Feng Y. (2018). Epigenetic regulation in B-cell maturation and its dysregulation in autoimmunity. *Cellular & Molecular Immunology*.

[23] Wei Y., Zhang F., Zhang Y. (2019). Post-transcriptional regulator Rbm47 elevates IL-10 production and promotes the immunosuppression of B cells. *Cellular & Molecular Immunology*.

[24] Elias R. D., Ma W., Ghirlando R., Schwieters C. D., Reddy V. S., Deshmukh L. (2020). Proline-rich domain of human ALIX contains multiple TSG101-UEV interaction sites and forms phosphorylation-mediated reversible amyloids. *Proceedings of the National Academy of Sciences of the United States of America*.

[25] Tollenaere M. A. X., Tiedje C., Rasmussen S. (2019). GIGYF1/2-driven cooperation between ZNF598 and TTP in posttranscriptional regulation of inflammatory signaling. *Cell Reports*.

[26] Duscha A., Gisevius B., Hirschberg S. (2020). Propionic acid shapes the multiple sclerosis disease course by an immunomodulatory mechanism. *Cell*.

[27] Wieckowski E., Wang G. Q., Gastman B. R., Goldstein L. A., Rabinowich H. (2002). Granzyme B-mediated degradation of T-cell receptor zeta chain. *Cancer Research*.

[28] Loeb C. R., Harris J. L., Craik C. S. (2006). Granzyme B proteolyzes receptors important to proliferation and survival, tipping the balance toward apoptosis. *The Journal of Biological Chemistry*.

[29] Han J., Goldstein L. A., Gastman B. R., Froelich C. J., Yin X. M., Rabinowich H. (2004). Degradation of Mcl-1 by granzyme B: implications for Bim-mediated mitochondrial apoptotic events. *The Journal of Biological Chemistry*.

[30] Shariati M., Meric-Bernstam F. (2019). Targeting AKT for cancer therapy. *Expert Opinion on Investigational Drugs*.

