# Supplementary material for: Short-Chain Fatty Acids Promote Immunotherapy by Modulating Immune Regulatory Property in B Cells
Source: J Immunol Res. 2021 Dec 10;2021:2684361. doi: 10.1155/2021/2684361 (PMC8683205; doi:10.1155/2021/2684361)
Supplement: Supplementary Materials — Supplemental materials include reagent information, a portion of experimental information and some results. Reagents and experimental procedures: skin prick tests, preparation of peripheral blood mononuclear cells and serum, high-performance liquid chromatography, cell culture, enzyme-linked immunosorbent assay, flow cytometry, isolation of immune cells, Western blotting, real-time quantitative RT-PCR, immunoprecipitation, mass spectrometry, and SIT. Supplemental figures: Figure S1: a schematic of mouse airway allergy model development. Figure S2: correlation coefficient between serum Th2 cytokines and serum AA or BA. Figure S3: assessment of immune suppressive effects of CD5+ B cells on T cell proliferation. Figure S4: knockdown of GPR41 or GPR43 in B cells. Figure S5: modulating TTP expression in B cells. Figure S6: GZMB amino acid sequence. [file 2684361.f1.docx]

**Supplemental materials**

**Reagents**

GPR41 and GPR 43 shRNA kits, antibodies (Ab) of TTP (Clone#: A-8), AKT1 (B-1), pAKT (B-5), T-bet (4B10), pSerine, CD19 (B-1, AF488), CD5 (H-3, AF546) and IL-10 (3C12C12, AF594) were purchased from Santa Cruz Biotech (Santa Cruz, CA). ELISA kits of IL-4, IL-5, IL-13, IL-10, sIgE, EPX and MCP1 were purchased from BioMart (Beijing, China). Naïve B cell isolation kit was purchased from Miltenyi Biotech (San Diego, CA). Vancomycin, neomycin, metronidazol, amphotericin-B, ampicillin, indole-3-PA and CpG were purchased from Sigma Aldrich (St. Louis., MO). Reagents and materials for RT-qPCR, Western blotting and immunoprecipitation was purchased from Invitrogen (Carlsbad, CA).

**Skin prick tests (SPT)**

SPT was performed for all AR patients and HC subjects. Allergens of SPT included aeroallergens, including mite mix, D. *farina*, D. *pteronyssinus*, mold mix, pollens (pine, poplar, rye, timothy grass, Bermuda grass, mugwort), animal dander (dog and cat). The allergens were purchased from Allergopharma (Germany). Histamine (10 mg/ml) and saline were used as positive and negative controls, respectively. If the mean wheal diameter was ≥3 mm larger than the negative control, the results were positive.

**Preparation of peripheral blood mononuclear cells (PBMCs) and serum**

Blood samples were obtained from each human subjects (20 ml per person) through the ulnar vein puncture. A portion of the sample was centrifuged (10,000 *g*; 4 °C) for 10 min. The serum was collected, and stored at -80 °C until use. PBMCs were separated from blood samples by the Percoll gradient density centrifugation, and cultured in RPMI1640 medium for further experiments.

**High performance liquid chromatography (HPLC)**

To determine the serum levels of AA, BA and PA, serum samples (or the standard AA, BA and PA) were analyzed by HPLC, that was performed using an Agilent TC-C18 column (250 × 4.6 mm, 5 μm; Agilent, MA, USA) at a column temperature of 35 °C using 0.05% (v/v) trifluoroacetic acid in DW (A) and acetonitrile (B) as a mobile phase, with the following gradient flow: 0-3 min, 20% B; 3-10 min, 20-40%B; 10-20 min, 40-90%B; 20 min-26 min, 90%B; 26 min-27 min, 90-20%B; 27 min-33 min, 20%B. The injection volume was 10 μl and flow rate was 1.0 ml/min. The levels of AA, BA and PA were calculated against the standard samples.

**Cell culture**

Immune cells were prepared from relevant experiments, and cultured in RPMI1640 medium. The medium was supplemented with 10% fetal calf serum, 100 U/ml penicillin, 0.1 mg/ml streptomycin, and 2 mM glutamine. Cell viability was greater than 99% as assessed by the Trypan blue exclusion assay. Less than 2% apoptotic cells (1.3 ± 0.33) were detected (by the annexin v and propidium iodide staining, and checked by FACS).

**Enzyme-linked immunosorbent assay (ELISA)**

Cytokine levels in the serum or culture supernatant were determined by ELISA with commercial reagent kits following the manufacturer’s instructions.

**Flow cytometry (FACS)**

Cells were collected from relevant experiments. In the surface staining, cells (10^6^ cells/sample) were stained with fluorescence-labeled antibodies (1 µg/ml) or isotype IgG for 30 min at 4 °C, washed with phosphate buffered saline (PBS), and analyzed with a flow cytometer (BD FACSCanto II). In the intracellular staining, cells were fixed with 1% paraformaldehyde (mixed with 0.05% Triton X-100 to promote the membrane permeability) for 1 h, washed with PBS, and then followed the procedures of the surface staining. The data were analyzed with a software Flowjo (TreeStar Inc., Ashland, OR) with the data obtained from isotype IgG as gating references.

**Isolation of immune cells**

Naive B cells were identified as those expressing CD19^+^, IgD^+^, and CD27^−^ {Payne, 2019 #8}. Naïve B cells and CD5^+^ B cells were isolated from PBMCs with the magnetic cell sorting reagent kits following the manufacturer’s instructions.

**Western blotting**

Cells were obtained from relevant experiments. Proteins were extracted from cells, fractioned by SDS-PAGE (sodium dodecyl sulfate polyacrylamide gel electrophoresis), and transferred onto a PVDF membrane. Non-specific binding of the membrane was blocked by incubating with 5% skim milk for 30 min. The membrane was then incubated with primary antibodies (detailed in figures) diluted at 100 ng/ml overnight at 4 °C, washed with TBST (Tris-buffered saline mixed with 0.05% Tween 20) 3 times, incubated with horseradish peroxidase-labeled second antibodies (diluted at 10 ng/ml) for 2 h at room temperature, washed with TBST 3 times. Immunoblots on the membrane were developed with the enhanced chemiluminescence, and photographed in an imaging device (UVP, Cambridge, UK).

**Real-time quantitative RT-PCR (RT-qPCR)**

Total RNA was extracted from cells collected from relevant experiments, and converted to cDNA with a reverse transcription kit following the manufacturer’s instructions. The products were amplified in a qPCR device (Bio-Rad CFX96) with the SYBR Green Master Mix in the presence of relevant primers, including IL-10 (human, ctctgttgcctggtcctcc and ctcgaagcatgttaggcagg; mouse, ataactgcacccacttccca and gggcatcacttctaccaggt), tristetraproline (TTP) (gactgagctatgtcggacct and ggttgtggatgaagtggcag), and granzyme B (tgacagtgcaggaagatcga and ataggagacaatgccctggg). The results were calculated with the 2^-∆∆Ct^ method against the housekeeping gene β-actin.

**Immunoprecipitation (IP)**

Proteins were extracted from cells obtained from relevant experiments, precleared by incubating with protein G coated agarose beads for 2 h to remove the pre-existing immune complexes. The samples were centrifuged at 13,000 *g* for 10 min. The supernatant was collected, and incubated with antibodies of interest (diluted at 1 µg/ml) overnight. The immune complexes in samples were precipitated by incubating with protein G agarose beads for 2 h. The beads were collected by centrifugation for 10 min at 13,000 *g*. IP products on the beads were eluted, and analyzed by Western blotting. For detecting the phosphor T-bet, as anti-pT-bet antibody was not available, after staining the membrane with antibody of T-bet, the membrane was processed by the peeling-re-blotting procedures and stained with an anti-p-serine antibody.

**Mass spectrometry (MS)**

IP products precipitated by TTP Ab were analyzed by MS to elucidate the components forming complex with TTP following established procedures (Liu et al. Dust-mite-derived protein disulfide isomerase suppresses airway allergy by inducing tolerogenic dendritic cells. J Biol Chem 2021;296:100585). Briefly, Protein samples were loaded on C18 nanoLC trap column and washed by Nano-RPLC Buffer A (0.1% FA, 2% ACN) at 2μl/min for 10 mins. An elution gradient of 5-35% acetonitrile in 90 mins gradient was used on an analytical. ChromXP C18 column with a spray tip. Data acquisition was performed with a Triple TOF 5600 System (AB SCIEX, USA) fitted with a Nanospray III source (AB SCIEX, USA) and a pulled quartz tip as the emitter (New Objectives, USA). Data were acquired using an ion spray voltage of 2.5 kV, curtain gas of 30 PSI, nebulizer gas of 5 PSI, and an interface heater temperature of 150 °C. For information dependent acquisition, survey scans were acquired in 250 ms with a 2+ to 5+ charge-state. The total cycle time was fixed to 2.5 s. Dynamic exclusion was set for ½ of peak width (18 s).

**SIT (Allergen specific immunotherapy)**

Following published procedures with modification (Leonard SA, Martos G, Wang W, Nowak-Węgrzyn A, Berin MC. Oral immunotherapy induces local protective mechanisms in the gastrointestinal mucosa. J Allergy Clin Immunol 2012; 129:1579-87) with modifications, SIT was administrated for AR mice starting one week after the last antigen challenge. Briefly, mice were fed with OVA at the doses of 1 mg (days 1 and 2), 5 mg (days 3 and 4), 10 mg (days 5–7), 25 mg (days 8 and 9), and 50 mg (days 10–14) with or without mixing with sema3A (0.2 mg/mouse daily).


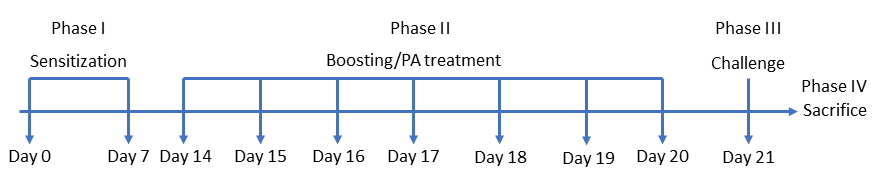


**Figure S1. A schematic of mouse airway allergy model development**. Phase I (sensitization): Mice were subcutaneously injected with ovalbumin (OVA, 100 μg/mouse mixed with 0.1 ml Alum) in the back skin on day 0 and day 7, respectively. Phase II (Boosting/PA treatment): Mice were boosted by nasal instillation with OVA (20 μl/nostril, 1 mg/ml) daily from day 14 to day 20. Phase III (challenge): Mice were challenged by nasal instillation with OVA (20 μl/nostril, 5 mg/ml) on day 21. After recording the clinical symptoms (nasal itch and sneezing), mice were sacrificed by cervical dislocation.


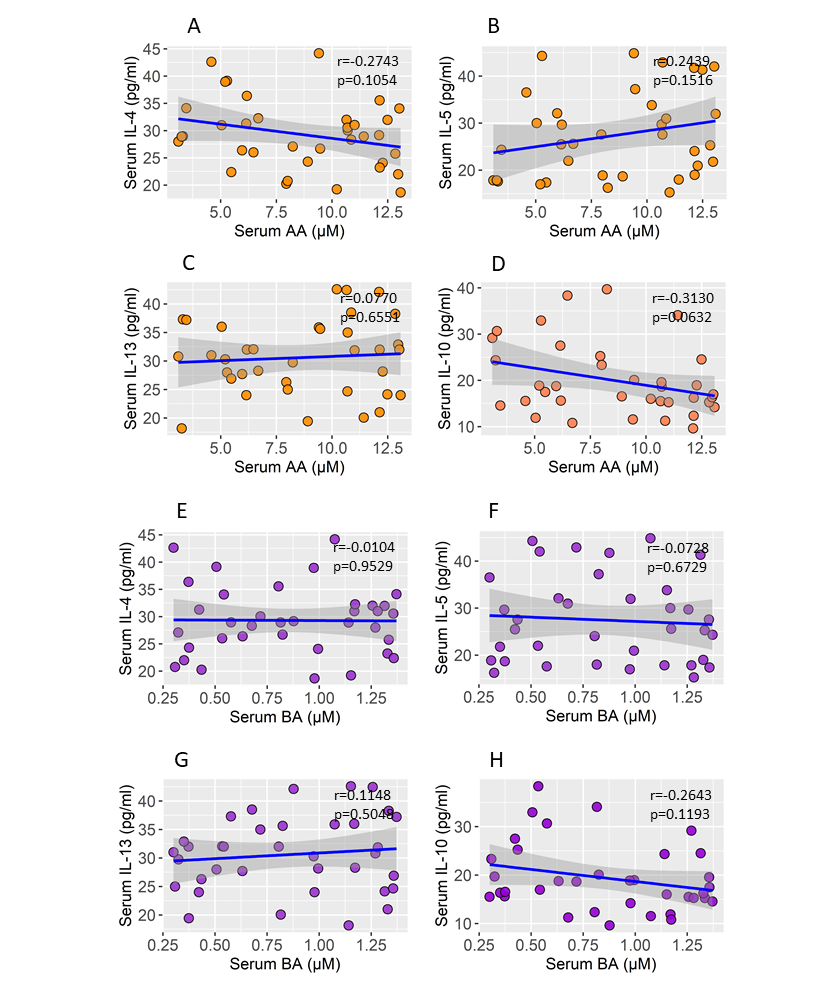


**Figure S2. Correlation coefficient between serum Th2 cytokines and serum AA or BA**. Blood samples were collected from 36 AR patients and 36 healthy control (HC) subjects. The serum was separated from blood samples and analyzed by HPLC and ELISA. A-F, correlation coefficient between serum AA (A-C) or BA (D-F) and serum Th2 cytokine and IL-10 levels of the AR group.


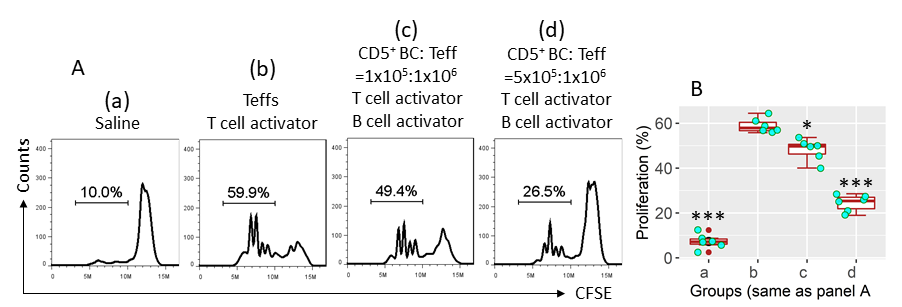


**Figure S3. Assessment of immune suppressive effects of CD5^+^ B cells on T cell proliferation**. CD5^+^ B cells and CD4^+^ CD25⁻ effector T cells (Teff, labeled with CFSE) were isolated from blood samples collected from HC subjects by magnetic cell sorting, and cultured in the conditions denoted above FACS panels. T cell activator: Anti-CD3 (2 µg/ml)/CD28 (5 µg/ml) Abs. B cell activator: LPS (1 µg/ml). Culture time: 3 days. A, gated FACS plots show proliferating T cells. B, boxplots show median (IQR) of proliferating T cell frequency from 6 experiments. *, p<0.05, ***, p<0.001 (ANOVA + Dunnett’s test), compared with group b.


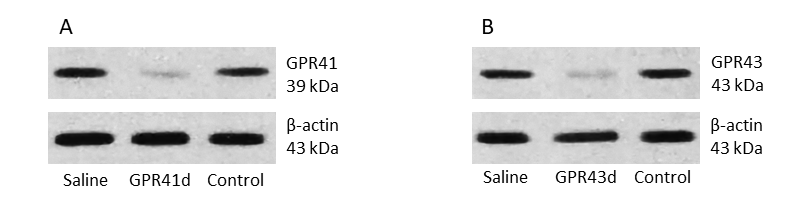


**Figure S4. Knockdown of GPR41 or GPR43 in B cells**. Human B cells were isolated from blood samples collected from HC subjects, and treated with shRNA kits of GPR41 or GPR43 or control reagents following the manufacturer’s instructions. A-B, immunoblots show GPR41 (A) or GPR43 (B) protein levels in B cells.


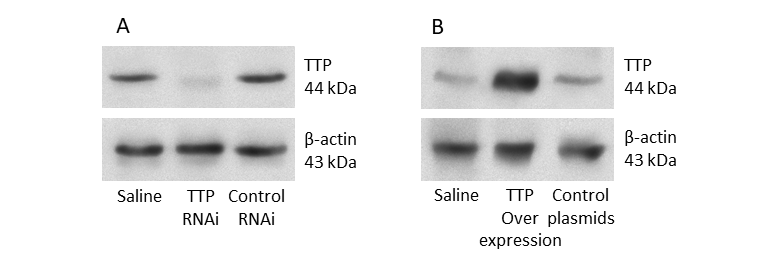


**Figure S5. Modulating TTP expression in B cells**. B cells were isolated from blood samples collected from HC subjects, and treated with TTP RNAi (A) or transfected with TTP-expressing plasmids (B) following the manufacturer’s instruction. The immunoblots show the TTP RNAi results (A) and the TTP overexpression results (B) in B cells.


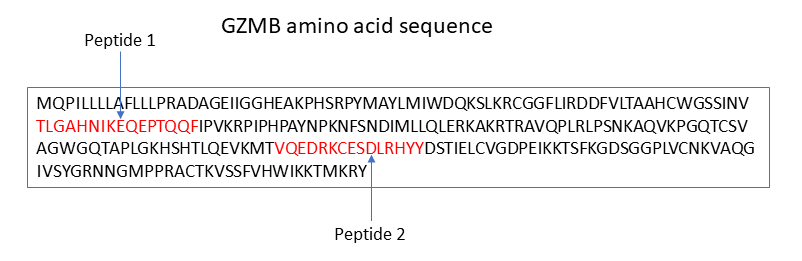


**Figure S6. GZMB amino acid sequence**. The GZMB sequence was cited from NCBI database (NM_004131.6). The highlighted peptides are the representative fractions identified by MS in IP products of B cells.
